# Perioperative inflammation in gynecologic oncology: nursing strategies for enhanced recovery and complication prevention

**DOI:** 10.3389/fmed.2026.1986451

**Published:** 2026-09-30

**Authors:** Xiaoyan Chen, Zhongshu Zhang, Shanli Xie, Jianping Liu, Ling Zhao, Yi Peng

**Affiliations:** 1 Department of Gynecology, Guangyuan First People’s Hospital, Guangyuan, Sichuan, China; 2 Guangyuan Lizhou Secondary Vocational and Technical School, Guangyuan, Sichuan, China; 3 Department of Pediatrics, Guangyuan First People’s Hospital, Guangyuan, Sichuan, China

**Keywords:** enhanced recovery after surgery, gynecologic oncology, nursing care, patient-reported outcomes, perioperative inflammation, postoperative complications, prehabilitation

## Abstract

Major gynecologic oncology surgery produces an acute inflammatory and neuroendocrine stress response superimposed on cancer-associated inflammation, frailty, malnutrition, anemia, and treatment-related physiologic vulnerability. Although this response is necessary for tissue repair, excessive or poorly resolved activation can contribute to pain, postoperative nausea and vomiting, ileus, insulin resistance, endothelial dysfunction, infection, venous thromboembolism, functional decline, and delayed return to oncologic treatment. Enhanced recovery after surgery (ERAS) pathways reduce avoidable stressors through coordinated, multimodal care, but their effectiveness depends on reliable implementation across the entire perioperative continuum. Nurses are positioned at the interfaces where risk is identified, pathway elements are delivered, trajectory deviations are recognized, and escalation occurs. This Mini Review synthesizes the inflammatory biology most relevant to gynecologic oncology nursing and translates it into practical strategies for preoperative screening and education, protocol verification, multimodal symptom control, early feeding and mobilization, infection and thromboembolism prevention, structured postoperative surveillance, and digitally supported follow-up. C-reactive protein and other inflammatory indices may complement assessment in selected pathways, but they are nonspecific and should not replace clinical evaluation or procedure-specific escalation criteria. The central proposition is that nursing care can be made inflammation-sensitive without becoming biomarker-driven: modifiable inflammatory inputs should be reduced, physiologic reserve should be preserved, self-reinforcing complication loops should be interrupted, and recovery trajectories should be monitored consistently. Future studies should test nurse-coordinated bundles using implementation, patient-reported, functional, and oncologic-process outcomes.

## 1 Introduction

Gynecologic oncology surgery ranges from minimally invasive staging procedures to prolonged open cytoreductive operations involving bowel, upper abdominal, urinary tract, or diaphragmatic procedures. The 2026 Enhanced Recovery After Surgery (ERAS) Society update frames perioperative care as a coordinated pathway rather than a collection of isolated postoperative tasks (1). This distinction is important for nursing practice because risk recognition, education, symptom control, mobilization, nutrition, device management, discharge preparation, and follow-up are distributed across settings and professional boundaries.

Inflammation is integral to both malignancy and surgical repair. Cancer-associated cytokine activity can coexist with anemia, catabolism, malnutrition, sarcopenia, metabolic disease, and reduced cardiopulmonary reserve. Surgery then initiates tissue injury, damage-associated molecular pattern signaling, cytokine release, oxidative stress, neuroendocrine activation, insulin resistance, and coagulation changes (2–5). These responses are adaptive when proportionate and self-limited. When amplified by extensive surgery, complications, immobility, uncontrolled symptoms, hypothermia, hyperglycemia, or infection, they can become a self-reinforcing stress state rather than a linear recovery process.

The perioperative period may also transiently suppress cell-mediated and natural killer cell function while activating pathways that support endothelial permeability and thrombosis. The relevance of these changes to long-term cancer outcomes remains biologically plausible but clinically difficult to separate from tumor biology, surgical complexity, residual disease, and treatment selection (6–8). Accordingly, the immediate nursing objective is not to claim direct prevention of recurrence through inflammation control, but to reduce preventable morbidity, preserve function, and support timely continuation of cancer treatment.

Gynecologic oncology ERAS recommendations were developed through successive preoperative, intraoperative, and postoperative guidelines and updated evidence syntheses (9–12). Early pathway studies and randomized trials demonstrated that standardized recovery programs can be implemented across laparotomy and mixed gynecologic surgical populations and can improve selected recovery outcomes without increasing readmission (13–18). Component-focused studies have further examined opioid exposure and patient-reported symptoms, protocol adherence, return to intended oncology treatment, perioperative nutrition, and gastrointestinal recovery (19–23). Building on this evidence, this Mini Review interprets perioperative inflammation through a nursing lens and proposes a practical, trajectory-based framework for complication prevention.

Review methodology. This targeted narrative Mini Review used PubMed/MEDLINE and Embase searches through August 2026. Search concepts combined gynecologic oncology surgery, ERAS, perioperative inflammation, nursing, postoperative complications, VTE, nutrition, prehabilitation, biomarkers, ePROs, and remote monitoring. We prioritized ERAS and oncology guidelines, randomized studies, systematic reviews or meta-analyses, and gynecologic oncology-specific clinical evidence. The review was not designed as a systematic review or meta-analysis, and no formal risk-of-bias grading was performed.

A concise evidence map separating proposed inflammatory/physiologic rationale from demonstrated clinical benefit is provided in Table 1.

**Table 1:** Relationship between selected perioperative interventions, proposed inflammatory/physiologic rationale, demonstrated clinical benefit, and evidence type.

Intervention/nursing action	Proposed rationale	Supported clinical benefit/use	Evidence
Multimodal analgesia/opioid sparing	Limits nociceptive and sympathetic stress	Lower opioid use and improved patient-reported recovery (19, 20)	ERAS cohort
Early oral feeding	Supports GI recovery and limits prolonged catabolism	Earlier feeding and GI recovery (22, 23)	RCT/clinical
Early mobilization	Limits deconditioning and immobilization-related VTE risk	Supports functional recovery; no universal step target (35)	Guideline/review
Infection-prevention bundle	Limits secondary inflammatory burden from infection	Prevention of avoidable surgical-site infection (36–39)	Guidelines
Mechanical+pharmacologic VTE prophylaxis	Addresses malignancy-, surgery-, and immobility-related thrombotic risk	Guideline-supported; extended prophylaxis for selected major cancer surgery (40–42)	Guideline/RCT
ePRO/telephone/wearable follow-up	Extends recovery-trajectory surveillance after discharge	Feasibility and earlier problem identification; major outcome benefit unproven (43–45)	Prospective/observational

## 2 Perioperative inflammatory biology relevant to nursing care

### 2.1 Baseline vulnerability before the incision

The perioperative inflammatory phenotype begins before surgery. Advanced tumor burden, ascites, prior systemic therapy, infection risk, anemia, hypoalbuminemia, and loss of muscle reserve can reduce the margin for physiologic compensation. In a pilot study of advanced ovarian cancer, inflammatory and nutritional markers including C-reactive protein (CRP), interleukin-6, albumin, and the Glasgow Prognostic Score were associated with perioperative morbidity or survival, but the small sample and observational design preclude use as standalone decision rules (24). CRP also follows a predictable postoperative rise after uncomplicated gynecologic surgery, emphasizing that a single elevated value may reflect expected tissue injury rather than infection (25). The clinically relevant signal is therefore a discordant trajectory: inflammatory laboratory trends that fail to resolve or rise together with fever, tachycardia, worsening pain, altered bowel function, respiratory deterioration, wound changes, or functional decline.

Nutrition, anemia, frailty, and sarcopenia are not interchangeable, but they frequently overlap and may amplify inflammatory and catabolic responses. Cancer and surgical nutrition guidelines support systematic screening and individualized nutrition care, while perioperative anemia guidance favors early identification and cause-directed management rather than reflexive transfusion (26–29). Frailty and nutritional vulnerability predict adverse outcomes after ovarian cancer surgery, and sarcopenia is associated with poorer survival across ovarian cancer cohorts (30, 31). For nurses, these findings justify structured screening and escalation, not exclusion from surgery based on one score.

### 2.2 The surgical stress-response hub

Incision, tissue handling, ischemia-reperfusion, blood loss, transfusion, and microbial exposure release endogenous danger signals and activate innate immune pathways. Toll-like receptor and nuclear factor-kappa B signaling promote interleukin-6, interleukin-1 beta, tumor necrosis factor-alpha, and chemokine production; the liver generates acute-phase proteins such as CRP; and reactive oxygen species increase. In parallel, hypothalamic-pituitary-adrenal and sympathetic activation promote catabolism and insulin resistance, while endothelial and platelet activation shift the hemostatic balance toward thrombosis. Extensive procedures may also produce transient postoperative immune dysfunction (2–8). Figure 1 integrates these pathways with the clinical complications that nurses are most likely to detect first.

**Figure 1:**
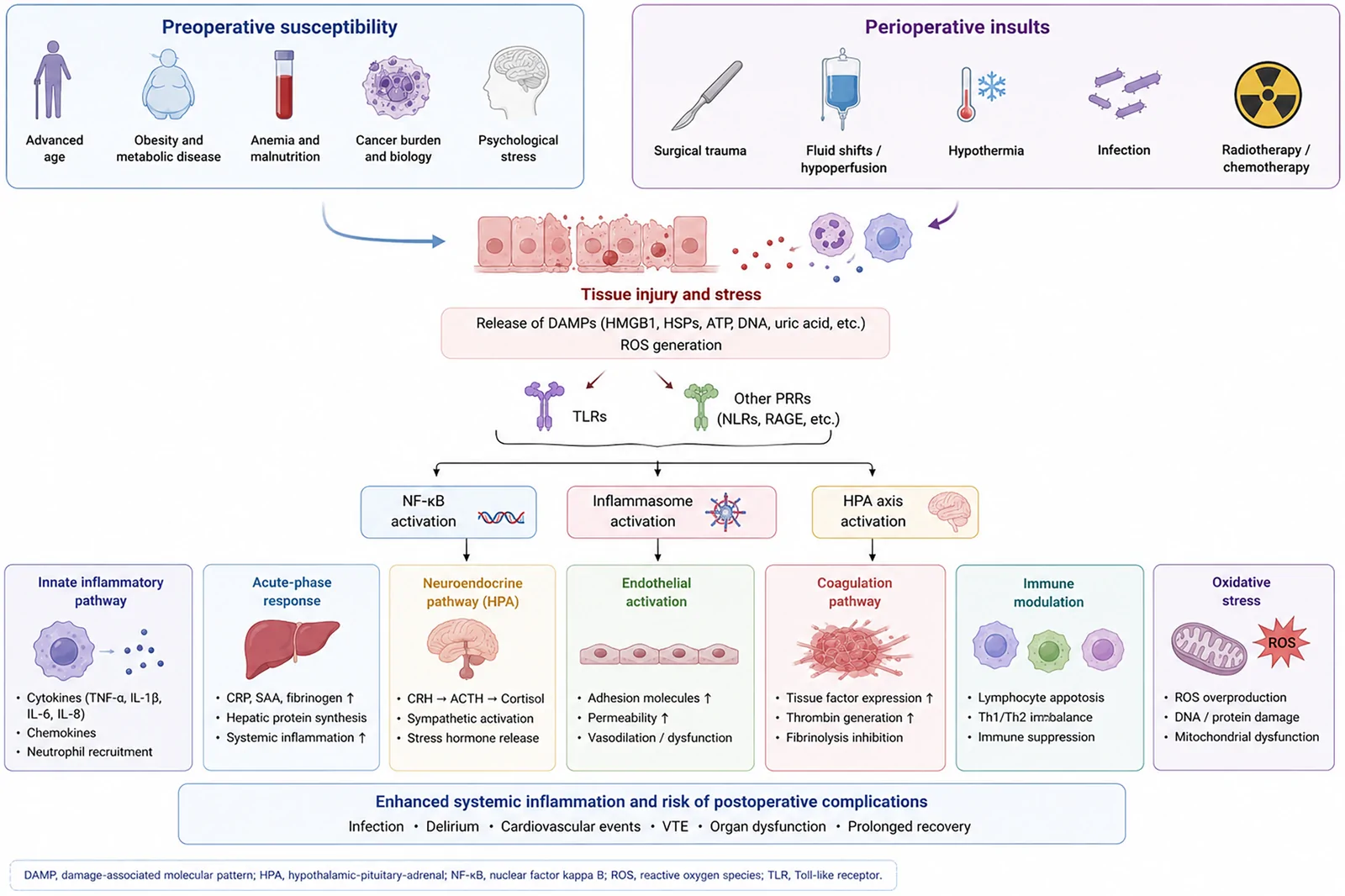
Perioperative inflammation-complication network in gynecologic oncology. Preoperative susceptibility and perioperative insults converge on innate inflammatory, acute-phase, neuroendocrine, endothelial, coagulation, and immune pathways. The resulting symptom, organ, infectious, and thromboinflammatory complications can feed back through pain and immobility. Nursing surveillance links symptoms, vital signs, wound and device findings, intake, mobility, and contextual laboratory trends to timely multidisciplinary escalation. DAMP, damage-associated molecular pattern; HPA, hypothalamic-pituitary-adrenal; NF-*κ*B, nuclear factor kappa B; ROS, reactive oxygen species; TLR, Toll-like receptor.

This biology should not be interpreted as a mandate to normalize every inflammatory marker. A transient response is necessary for hemostasis, host defense, and wound repair. The practical objective is to limit unnecessary inflammatory inputs and identify deviation from the expected recovery trajectory. Procedure type, operative duration, blood loss, transfusion, baseline comorbidity, and postoperative day must therefore accompany any interpretation of symptoms or laboratory results.

## 3 From inflammation to postoperative morbidity

Pain, nausea, ileus, and immobility can form an early feedback loop. Tissue inflammation lowers nociceptor thresholds; pain and anxiety increase sympathetic activation; opioids may worsen nausea, sedation, and gastrointestinal dysmotility; and immobility accelerates deconditioning and thrombotic risk. ERAS programs that use multimodal analgesia and structured symptom assessment can reduce opioid exposure and improve patient-reported recovery (19, 20). Current postoperative nausea and vomiting guidance emphasizes risk assessment, multimodal prophylaxis, rescue treatment from a different drug class, and attention to hydration and opioid burden (34). Early mobilization is a core recovery element, although protocols vary and evidence is stronger for reducing immobilization-related risk and supporting function than for a single universal step target (35).

The early inflammatory response to uncomplicated surgery overlaps with the first manifestations of infection. Fever, leukocytosis, pain, and CRP elevation are therefore nonspecific in isolation. Surgical-site infection prevention depends on a bundle that begins before incision and continues through wound care: appropriate antibiotic selection and timing, skin preparation, normothermia, glycemic management, aseptic device care, and prompt evaluation of wound or systemic deterioration (36–39). Serial CRP may support selected local pathways, but routine escalation should be based on the total clinical picture and procedure-specific concerns, including occult bowel, urinary tract, or anastomotic complications (25).

Gynecologic malignancy, major pelvic or abdominal surgery, immobility, inflammation, and patient-specific risk factors converge to increase venous thromboembolism (VTE) risk. Evidence-based pathways combine mechanical prophylaxis, timely inpatient pharmacologic prophylaxis when hemostasis is secure, early ambulation, and extended post-discharge prophylaxis for selected high-risk major cancer operations (40, 41). A randomized trial in women undergoing surgery for gynecologic malignancy found oral apixaban to be a feasible option for postoperative prophylaxis (42), but it should be considered within guideline-based patient selection rather than as a universal replacement for low-molecular-weight heparin. Bleeding risk, renal function, drug interactions, adherence, and peri-neuraxial timing remain prescriber-led considerations; nurses support safe administration, compression-device use, mobility, bleeding surveillance, education, and follow-up.

Complications also have oncologic-process consequences. Prolonged ileus, infection, VTE, nutritional decline, or loss of functional reserve can extend hospitalization, increase readmission, and delay intended chemotherapy. Higher ERAS compliance and pathway implementation have been associated with better perioperative outcomes and earlier return to intended oncologic treatment after interval cytoreduction (20, 21).

## 4 Nursing strategies across the perioperative continuum

Core principles are broadly applicable, but recovery targets require procedure-specific calibration. Minimally invasive staging generally produces a lower inflammatory burden and faster gastrointestinal and functional recovery than major open cytoreduction with bowel, diaphragmatic, splenic, urinary tract procedures, or HIPEC. CRP trajectories, fluid goals, mobility targets, nutritional support, infection surveillance, and expected length of stay should therefore be interpreted against procedural magnitude.

### 4.1 Pre-admission assessment, optimization, and teach-back education

Preoperative nursing assessment should translate broad surgical risk into actionable pathway needs. A practical, author-proposed assessment framework should address recent weight loss and intake, frailty and mobility, anemia, glycemic control, smoking, cardiopulmonary symptoms, medication and anticoagulant use, prior treatment toxicities, VTE history, cognition, health literacy, caregiver availability, transportation, and digital access. This is not a validated minimum dataset; positive screens should prompt referral to the appropriate surgical, anesthesia, nutrition, rehabilitation, pharmacy, or medical team. This approach aligns with nutrition, patient blood management, and frailty evidence while avoiding the false precision of a single laboratory or screening score (26–31).

Multimodal prehabilitation can combine aerobic and resistance activity, respiratory training, nutrition support, psychological preparation, and optimization of comorbidity. Evidence in gynecologic oncology is emerging and remains heterogeneous, but early studies support feasibility and potential improvement in perioperative readiness (32). Education should establish concrete postoperative expectations: oral intake, analgesia, nausea prevention, mobilization, respiratory exercises, catheter and drain plans, VTE prophylaxis, discharge criteria, and warning signs. Teach-back is preferable to passive information delivery because it exposes misunderstandings and can improve mobilization-related outcomes after gynecologic surgery (33).

### 4.2 Operating room and post-anesthesia care interfaces

Although many intraoperative interventions are prescribed or delivered by anesthesia and surgery teams, nursing verification determines whether the bundle is reliable. High-value checks include antibiotic timing and redosing cues, availability and use of mechanical VTE prophylaxis, warming measures, glucose and fluid plans, pressure-injury prevention, specimen and device documentation, and an explicit plan for postoperative analgesia and antiemetic prophylaxis (1, 9, 34, 36–39). The transfer from the operating room to the post-anesthesia care unit and ward should communicate procedure extent, blood loss and transfusion, unexpected contamination or injury, airway and oxygen needs, regional blocks, drains and stomas, urine output, mobility restrictions, and individualized escalation triggers.

In the immediate recovery phase, repeated assessment is more informative than a single score. Nurses should trend consciousness, respiratory status, temperature, hemodynamics, bleeding, urine output, pain, nausea, motor function, and early signs of delirium. Failure to achieve expected stabilization should prompt structured escalation rather than repeated symptomatic treatment without diagnostic reconsideration.

### 4.3 Multimodal symptom control, early feeding, and mobilization

Pain management should combine regular assessment with protocolized non-opioid analgesia, regional techniques where used, and opioids reserved for breakthrough pain or contraindications to other agents. The assessment must distinguish expected incisional pain from pain that is increasing, diffuse, pleuritic, associated with peritonism, or accompanied by altered vital signs. Nausea control should be linked to oral intake, hydration, mobilization, and medication review rather than treated as an isolated symptom (19, 23, 34). Sedation, dizziness, orthostatic intolerance, and respiratory depression require active surveillance because they can obstruct every subsequent recovery element.

Early oral intake and mobilization should be individualized but not left to chance. Nursing workflows can specify first-drink, first-meal, chair, standing, and walking goals; document barriers; and trigger support from physiotherapy, nutrition, pharmacy, or the medical team. The relevant endpoint is progression in function, not completion of a checkbox. Recurrent vomiting, escalating abdominal distension, absent recovery of gastrointestinal function, new hypoxemia, or inability to mobilize should prompt reassessment for ileus, obstruction, infection, bleeding, cardiopulmonary events, or medication effects (22, 23, 35). Sleep protection, orientation, daylight exposure, hearing or visual aids, and removal of unnecessary lines can further reduce delirium and functional decline.

### 4.4 Infection, wound, device, glycemic, and VTE prevention

Postoperative prevention bundles should be visible in nursing documentation. Wound and stoma assessment, aseptic catheter and drain care, early removal of unnecessary devices, oral hygiene, respiratory exercises, glycemic surveillance, normothermia, pressure-injury prevention, and mobilization each target a different link in the complication network (36–39). A change in wound appearance, drainage, odor, pain, or separation should be photographed or measured according to local policy and escalated with associated systemic findings. Persistent tachycardia, oliguria, oxygen requirement, confusion, or reduced mobility may precede overt fever and should not be attributed automatically to pain or anxiety.

For VTE prevention, nurses should confirm that mechanical devices are fitted and functioning, pharmacologic doses are administered or deliberately withheld with a documented rationale, ambulation is progressive, and patients understand the duration and technique of post-discharge prophylaxis. New unilateral swelling, chest pain, dyspnea, hemoptysis, unexplained tachycardia, syncope, or a sudden decline in exercise tolerance requires urgent evaluation (40–42). Bleeding surveillance is equally important and includes wound, vaginal, urinary, gastrointestinal, and occult signs.

### 4.5 Trajectory-based surveillance and escalation

Inflammation-sensitive monitoring is not synonymous with frequent laboratory testing. It means defining an expected recovery trajectory and detecting divergence across domains: symptoms, vital signs, oxygenation, mental status, wound and device findings, intake, bowel and bladder function, mobility, sleep, and laboratory data when ordered. CRP, white blood cell count, hemoglobin, creatinine, glucose, and lactate must be interpreted in context. IL-6, procalcitonin, and NLR/PLR are selective or investigational adjuncts rather than routine requirements across gynecologic oncology ERAS pathways. Postoperative D-dimer is nonspecific after major cancer surgery and should not be used as a stand-alone routine VTE surveillance test. A patient whose CRP is elevated but clinically improving differs from a patient with a rising CRP, worsening pain, tachycardia, reduced intake, and new oxygen need (24, 25).

### 4.6 Discharge transition, ePROs, and remote follow-up

Earlier discharge transfers surveillance responsibility to patients and caregivers. Discharge readiness should include stable symptoms on an oral regimen, adequate intake and mobility for the home environment, a clear bowel and bladder plan, wound and device competence, medication reconciliation, VTE prophylaxis teaching, contact pathways, and comprehension verified by teach-back. Written instructions should distinguish expected symptoms from urgent red flags and should specify who will respond after hours.

Electronic patient-reported outcomes (ePROs), telephone follow-up, wearable activity data, and mobile engagement tools can extend recovery monitoring beyond hospital walls. Prospective studies in gynecologic oncology support feasibility and the potential for earlier identification of postoperative problems (43–45), but evidence that these technologies independently reduce major morbidity, readmission, or mortality remains limited. These systems should not simply generate alerts. They require defined ownership, response times, escalation algorithms, documentation, and contingency plans for patients with limited connectivity, language barriers, disability, or low digital confidence.

## 5 Implementation priorities and evidence gaps

The effectiveness of an inflammation-sensitive pathway depends more on reliability than on the number of elements listed. A nurse coordinator or designated pathway lead can support staff education, standard work, daily goal review, discharge readiness, and audit. This emphasis on implementation fidelity is consistent with the 2023 ERAS Society update, which specifically addressed barriers to adherence and pathway implementation (46). Useful process measures include screening completion, antibiotic and prophylaxis timing, temperature control, opioid exposure, time to oral intake and mobilization, catheter duration, prophylaxis adherence, ePRO response time, and reasons for pathway deviation. Outcomes should include complications, length of stay, readmission, patient-reported symptom burden, functional recovery, equity, and time to intended oncologic treatment (1, 12, 15, 20, 21).

Several evidence gaps remain. Direct causal evidence that each ERAS or nursing component improves outcomes specifically by attenuating inflammation is limited; most benefits arise from multimodal pathways. Biomarker thresholds are not standardized across minimally invasive surgery, laparotomy, cytoreduction, and hyperthermic intraperitoneal chemotherapy, and CRP trajectories are influenced by procedure magnitude and baseline inflammation. Prehabilitation trials remain heterogeneous, and digital monitoring studies are often single-center feasibility projects. As a targeted narrative review, literature selection may introduce selection and publication bias, and no formal evidence grading or risk-of-bias assessment was performed. Future pragmatic trials should evaluate nurse-coordinated bundles in diverse settings, prespecify escalation rules, capture implementation fidelity, and test whether combining clinical trajectories with parsimonious biomarkers improves detection without increasing unnecessary testing or alarm fatigue.

## 6 Discussion and conclusion

Perioperative inflammation provides a coherent explanation for why apparently separate nursing tasks are interdependent. Uncontrolled pain can impede breathing and movement; nausea can delay nutrition and mobilization; immobility can intensify deconditioning and VTE risk; and infection or bleeding can amplify the inflammatory stress response. The nursing contribution is therefore not a single anti-inflammatory intervention. It is the reliable coordination of measures that reduce avoidable stress, preserve physiologic reserve, interrupt complication loops, and identify deterioration early.

In gynecologic oncology, this approach should remain protocolized, multidisciplinary, and trajectory based. Biomarkers may support but cannot replace clinical assessment. The strongest current strategy is to embed nursing surveillance and escalation within evidence-based ERAS pathways, extend monitoring safely into the home, and measure recovery in terms of symptoms, function, complications, equity, and timely access to subsequent cancer treatment.
